# Arsenic Mobilization and Transformation by Ammonium-Generating Bacteria Isolated from High Arsenic Groundwater in Hetao Plain, China

**DOI:** 10.3390/ijerph19159606

**Published:** 2022-08-04

**Authors:** Zhou Jiang, Xin Shen, Bo Shi, Mengjie Cui, Yanhong Wang, Ping Li

**Affiliations:** 1 School of Environmental Studies, China University of Geosciences, Wuhan 430074, China; 2 State Key Laboratory of Biogeology and Environmental Geology, China University of Geosciences, Wuhan 430074, China

**Keywords:** arsenic, DNRA, N-fixation, methylation, demethylation, groundwater

## Abstract

Arsenic (As) mobilization in groundwater involves biogeochemical cycles of carbon, iron, and sulfur. However, few studies have focused on the role of nitrogen-metabolizing bacteria in As mobilization, as well as in the transformation between inorganic and organic As in groundwater. In this study, the nitrogen and As metabolisms of *Citrobacter* sp. G-C1 and *Paraclostridium* sp. G-11, isolated from high As groundwater in Hetao Plain, China, were characterized by culture experiments and genome sequencing. The results showed *Citrobacter* sp. G-C1 was a dissimilatory nitrate-reducing bacterium. The dissimilatory nitrate reduction to ammonia (DNRA) and As-detoxifying pathways identified in the genome enabled *Citrobacter* sp. G-C1 to simultaneously reduce As(V) during DNRA. *Paraclostridium* sp. G-11 was a nitrogen-fixing bacterium and its nitrogen-fixing activity was constrained by As. Nitrogen fixation and the As-detoxifying pathways identified in its genome conferred the capability of As(V) reduction during nitrogen fixation. Under anaerobic conditions, *Citrobacter* sp. G-C1 was able to demethylate organic As and *Paraclostridium* sp. G-11 performed As(III) methylation with the *arsM* gene. Collectively, these results not only evidenced that ammonium-generating bacteria with the *ars* operon were able to transform As(V) to more mobile As(III) during nitrogen-metabolizing processes, but also involved the transformation between inorganic and organic As in groundwater.

## 1. Introduction

Arsenic (As) is a toxic metalloid and has many species in the environment, such as inorganic As(III) and As(V), organic monomethylarsonic acid (MMA), dimethylarsinic acid (DMA), trimethylarsinic acid (TMA), and arsenobetaine (AsB). Long-term exposure to As can cause disorders of the skin, vascular and nervous systems, as well as cancer. Naturally occurring As in groundwater threatens the health of hundreds of millions of people worldwide, with hot spots present in Argentina, Mexico, the United States, India, Bangladesh, Myanmar, Cambodia, Vietnam, and China [1]. The mechanisms of As enrichment in groundwater mainly include (1) the reductive dissolution of As-bearing iron oxides under reducing conditions, (2) the oxidative dissolution of As-bearing sulfide minerals under oxidizing conditions, and (3) the competitive adsorption between As and phosphate/bicarbonate under alkaline conditions [2,3,4]. The first mechanism prevails in late Pleistocene-Holocene aquifer systems, such as fluvial-lacustrine basins and deltas from As-contaminated countries, and involves various metabolic processes of iron, sulfur, and carbon [2]. For example, iron oxide reduction by dissimilatory iron-reducing bacteria can directly release absorbed As into groundwater [5,6,7]. Sulfate reduction by dissimilatory sulfate-reducing bacteria generates sulfide, which can abiotically reduce As-bearing iron oxides, to desorb As or further sequester As through co-precipitation and adsorption on derived sulfide minerals [8,9,10,11]. The desorbed As(V) from iron oxides are reduced to more mobile As(III) by dissimilatory As-reducing bacteria containing *arrAB* operon or detoxifying As-reducing bacteria containing *ars* operon [12,13,14]. Carbon degradation by fermentation and methanogenesis provides labile carbon sources and electron donors for these functional microbes to facilitate As mobilization [15].

Ammonium in groundwater is often positively correlated with the As content, such as in the Hetao Plain, China [16], the Ganges River floodplain, Bangladesh [17], the Red River floodplain, Vietnam [18], and the Mekong Delta, Cambodia [19]. Organic matter degradation, as well as organic waste disposal, were thought to be the primary source of ammonium in groundwater [20,21]. Multi-isotopic investigations of nitrogen species in As-contaminated groundwater of Hetao Plain and Choushui River alluvial fan, China, revealed these nitrogen-metabolizing activities, including ammonification, dissimilatory nitrate reduction to ammonia (DNRA), nitrogen fixation, anammox and Feammox, and highlighted their potential roles in ammonium elevation and As mobilization [22,23]. These metabolic processes in groundwater were supported by the microbial community composition and functional potential, as shown by 16S RNA gene high-throughput sequencing, q-PCR, and Geochip [16,24,25]. Moreover, our previous metagenomic study on groundwater further proposed that ammonium-producing bacteria with *ars* operon, such as dissimilatory nitrate-reducing bacteria and nitrogen-fixating bacteria, may mediate the transformation from desorbed As(V) to more mobile As(III) in groundwater during respective nitrogen-metabolizing process [26]. However, these inferences about the role of ammonium-producing bacteria in As mobilization, based on geochemical and microbial ecological studies, need to be verified with pure stain culture experiments.

Apart for inorganic As, methylarsenicals including MMA and DMA are also detected in global high-As groundwater systems, accounting for about 0.1–10% of the total dissolved As content [27]. Laboratory cultivation experiments of high As sediments from southern Willamette Basin, USA [27] and Jianghan Plain, China [28] highlighted that As methylation played an important role in the As cycle in aquifers. Arsenic methylation is mediated by microorganisms containing *arsM*, with MMA, DMA, and TMA as the end products from As(III) [29]. Moreover, organic As is also demethylated to As(III) by microorganisms containing *arsI*, which encodes a nonheme-iron-dependent dioxygenase with C-As lyase activity [30]. Being major contributors to inorganic As mobilization, some iron-reducing bacteria, sulfate-reducing bacteria, and methanogens were also reported to involve a transformation between inorganic and organic As through methylation and demethylation. For example, *Shewanella oneidensis* MR-1 [31], *Geobacter metallireducens* GS-15 [32], *Clostridium* sp. BXM [33], *Methanosarcina acetivorans* C2A [34], and *Methanosarcina mazei* Gö1 [35] were able to methylate inorganic As(III) to organic MMA, DMA, and TMA. In addition, *Shewanella putrefaciens* CN32 [36], *Shewanella putrefaciens* 200 [37] and methanogen enrichment products from paddy soils [38] could demethylate organic As to inorganic As(III). Groundwater with a high As level harbored diverse phylogenetically As-methylating populations [39]. However, whether ammonium-generating bacteria in groundwater conduct As methylation and demethylation remains unclear.

Two detoxifying As(V)-reducing bacteria, *Citrobacter* sp. G-C1 and *Paraclostridium* sp. G-11, were isolated from high-As groundwater, Hetao Plain, Inner Mongolia, China [40]. Given this nitrogen and As metabolism, the objective of this study was therefore to (i) characterize ammonium production process of these two strains and verify the simultaneous As reduction during ammonium production; (ii) determine their potentials for As methylation and demethylation; and (iii) identify the key functional genes responsible for nitrogen and As metabolism by genome sequencing. We found that these two ammonium-generating bacteria with *ars* operon were not only able to reduce As(V) to more mobile As(III) during nitrogen-metabolizing processes, but also involved the transformation between inorganic and organic As.

## 2. Materials and Methods

### 2.1. Strains

*Citrobacter* sp. G-C1 and *Paraclostridium* sp. G-11 were isolated from high As groundwaters, Hetao Plain, Inner Mongolia, China. Our previous study indicated that *Citrobacter* sp. G-C1 is a facultative anaerobe and *Paraclostridium* sp. G-11 is an obligate anaerobe [40]. Both of them could reduce As(V) to As(III) when anaerobically incubated in chemically-defined medium (CDM) supplemented with 20 mM lactate and 1 mM As(V). The CDM (1 L) contained the following: 0.225 g K_2_HPO_4_, 0.225 g KH_2_PO_4_, 0.46 g NaCl, 0.225 g (NH_4_)_2_SO_4_, 0.117 g MgSO_4_•7H_2_O, 1 g yeast extract, 4.2 g NaHCO_3_, 1 mL trace element solution, and 10 mL vitamin solution [41].

### 2.2. DNRA by Citrobacter sp. G-C1 and Nitrogen Fixation by Paraclostridium sp. G-11

For the anaerobic culture experiments, medium was boiled and purged by N_2_ gas (purity ≥ 99.999%) to eliminate dissolved oxygen, unless otherwise stated. The medium was then dispensed into acid-cleaned serum bottles, sealed with butyl rubber stoppers and covered with aluminum foil, and autoclaved at 121 °C for 20 min. To determine DNRA activity, *Citrobacter* sp. G-C1 was inoculated into 10 mL LB medium and aerobically grown to the late-log phase. The cells were harvested by centrifugation at 8000× *g* for 3 min and washed three times with CDM. With 1% (*v*/*v*) inoculum, the cell suspension was added to 100 mL CDM supplemented with 10 mM or 20 mM lactate and 10 mM nitrate in an anaerobic glove box. Medium without bacterial cells was set as a control. All experiments were performed in triplicate and anaerobically incubated for 48 h at 37 °C. Aliquots of the culture medium were taken at 0, 4, 8, 12, 24, and 48 h and determined for nitrogen species and OD_600_.

The nitrogen-fixing activity of *Paraclostridium* sp. G-11 was tested using an acetylene reduction assay and ^15^N_2_ assimilation method in Burk’s N-free medium [42]. In this study, Burk’s N-free medium was supplemented with 20 mM lactate as an additional carbon source [43]. Briefly, *Paraclostridium* sp. G-11 was inoculated into 10 mL LB medium and grown anaerobically to the late-log phase. Cells were harvested by centrifugation at 8000× *g* for 3 min and washed three times with Burk’s N-free medium. The cell suspension (1 mL) was added to 30 mL Burk’s N-free medium. Medium without bacterial cells was set as a control. Then, 9.5 mL acetylene gas was injected into the headspace of each serum bottle and incubated at 37 °C. A 100-μL gas sample in each serum bottle was collected daily to determine the ethylene content using gas chromatography (GC-4000A, EWAI, Beijing, China). For ^15^N_2_ assimilation experiments, 30 mL headspace N_2_ gas in each serum bottle was drawn using a gas-tight syringe and replaced with 30 mL ^15^N_2_ gas. After 3 days of incubation at 37 °C, the microbial biomass was collected and lyophilized. The dried samples were manually pulverized and sent to Guangdong Ocean University for ^15^N content analysis by an element analyzer coupled with a stable isotope ratio mass spectrometer (EA Isolink-253 Plus, Thermo Fisher Scientific, Waltham, MA, USA) [44]. All experiments were performed in triplicate.

### 2.3. As(V) Reduction Co-Occurred with DNRA by Citrobacter sp. G-C1 and Nitrogen Fixation by Paraclostridium sp. G-11

Given the ability of ammonium generation and As(V)-detoxifying reduction, these two stains were used to verify the hypothesis that ammonium-producing bacteria with *ars* operon in groundwater are able to reduce As(V) to more mobile As(III) during their respective nitrogen metabolic processes [26]. For the As(V) reduction experiment co-occurring with DNRA, *Citrobacter* sp. G-C1 was pre-cultured in LB medium to the late-log phase, harvested by centrifugation, and washed three times with CDM. With 1% (*v*/*v*) inoculum, the cell suspension was added to 100 mL CDM supplemented with 20 mM lactate and the following: 10 mM nitrate; 10 mM nitrate and 100 µM As(V); 10 mM nitrate, 100 µM As(V) and 1 mM ferrous iron (Fe(II)); and 10 mM nitrate, 100 µM As(V), and 3 mM Fe(II). The addition of Fe(II) was to simulate the effect of Fe(II) in groundwater on DNRA and As(V) reduction. Medium containing 10 mM nitrate and 100 µM As(V) without bacterial cells was set as a control. All experiments were performed in triplicate and anaerobically incubated for 48 h at 37 °C. Aliquots of the culture medium were taken at 0, 4, 8, 12, 24, and 48 h and determined for As, Fe, and nitrogen species and OD_600_.

To test the As(V) reduction occurring concurrently with nitrogen fixation, Burk’s N-free medium supplemented with 100 µM As(V) was purged by argon (Ar) and N_2_ gases, respectively. Then 30-mL headspace gas in the N_2_ gas-purged serum bottle was replaced with 30 mL ^15^N_2_ gas. Following the same procedure as in the previous ^15^N_2_ assimilation experiment, 9.5 mL acetylene gas was injected into the headspace of each serum bottle and incubated at 37 °C. All experiments were performed in six replicates. A 100 μL gas sample and aliquots of the culture medium in each serum bottle were collected daily to determine ethylene content and As species analysis. After 3 days of incubation, triplicates of six were used to collect microbial biomass and determine the ^15^N content, as described above.

### 2.4. As Demethylation and Methylation

For the As demethylation experiment, these two stains were respectively inoculated into 10 mL LB medium and cultivated to the late-log phase. With 1% (*v*/*v*) inoculum, each cell suspension was added to 100 mL fresh LB medium supplemented with 1 μM MMA. Medium without bacterial cells was set as a control. All experiments were performed in triplicate and incubated for 9 days at 37 °C under aerobic and anaerobic conditions. Aliquots of the culture medium were taken at 0, 1, 2, 3, 5, 7, and 9 days and analyzed for As species.

For the As methylation experiment, each cell suspension added 1% (*v*/*v*) inoculum to 100 mL fresh LB medium supplemented with the following: 1 μM As(III); 1 μM As(III) and 5 mg/L S-adeno-sylmethionine (SAM); 5 μM As(III); 5 μM As(III) and 5 mg/L SAM; 10 μM As(III); 10 μM As(III) and 5 mg/L SAM. SAM addition was to determine the effect of the methyl donor on As biomethylation. Medium without bacterial cells was set as a control. All experiments were performed in triplicate and incubated for 28 days at 37 °C under anaerobic conditions. Aliquots of the culture medium were taken at 0, 0.5, 1, 6, 14, 23, and 28 days and centrifuged at 10,000× *g* for 10 min. The supernatant was used to determine the concentrations of extracellular As species. The cell pellets were washed with 10 mL 0.1 M KH_2_PO_4_/K_2_HPO_4_ buffer (pH 7.0) and 10 mL ultrapure water, to remove extracellular As, respectively. The cells were then sonicated in an ultrasonic cell disruptor until the cell suspension became clear [31]. After ultrasonication, the supernatants were filtered through 0.22-μm filter to analyze the intracellular As species.

### 2.5. Genome Sequencing and Functional Analyses

The strains were cultivated in LB medium and pelleted by centrifugation at 6000× *g* for 10 min. Genomic DNA was extracted from cell pellets using a DNeasy PowerSoil Kit (Qiagen, Valencia, CA, USA). DNA concentration and purity were measured using Qubit 3.0 fluorometer (Thermo Fisher Scientific, Waltham, MA, USA). Sequencing libraries were generated using an NEB Next^®^ UltraTM DNA Library Prep Kit by Illumina^®^ (New England Biolabs, MA, USA), following manufacturer’s recommendations. The library quality was assessed on a Qubit 3.0 and Agilent 4200 (Agilent, Santa Clara, CA, USA) system and then sequenced on an Illumina HiSeq X Ten platform at Guangdong Magigene Biotechnology Co., Ltd. (Shenzhen, China). About 1 Gbp (2 × 150 bp) reads for each genome were generated.

The raw sequencing data were trimmed to produce clean data using Trimmomatic and assembled using MEGAHIT. The assembled scaffold (≥500 bp) was used for ORF prediction with Prodigal, and those ORFs with a length of <90 bp were omitted. Functional annotation was performed by blasting ORFs to the KEGG database (http://www.kegg.jp/kegg/ (accessed on 15 May 2021)) using DIAMOND software with an e value threshold of 1 × 10^−10^. For each gene’s blast result, the best hit with lowest e value and a Blast coverage ratio between the query and reference sequences of >40 was chosen. Operon-mapper was used to predict the operons of these two genomes [45].

### 2.6. Determination of As, Iron, and Nitrogen Species

Arsenic species including As(III), As(V), MMA, and DMA were determined by high performance liquid chromatography combined with hydride generation atomic fluorescence spectrometry (HPLC-HG-AFS, Haiguang, Beijing, China) [46]. The concentrations of nitrate and nitrite were analyzed with an ECO IC ion chromatography (IC) (Metrohm AG, Herisau, Switzerland). Ammonium content was measured with the Nessler method [47]. Briefly, seignette reagent and Nessler reagent (0.5 mL each) were added to 25 mL diluted sample solution. The solution was agitated and left to stand for 10 min, and then measured for absorbance at 420 nm. The concentrations of Fe(II) and total Fe were determined spectrophotometrically (Spectramax 190, Molecular Devices, San Jose, CA, USA) using a ferrozine assay. The amount of Fe(III) was calculated as the difference between the total Fe and Fe(II) concentrations [48].

### 2.7. Sequence Data Deposition

The raw sequencing data of draft genomes of *Citrobacter* sp. G-C1 and *Paraclostridium* sp. G-11 were deposited to the Short Read Archive database at the NCBI (Accession number: PRJNA754837).

## 3. Results

### 3.1. Identification of DNRA and Nitrogen Fixation

**Figure 1:**
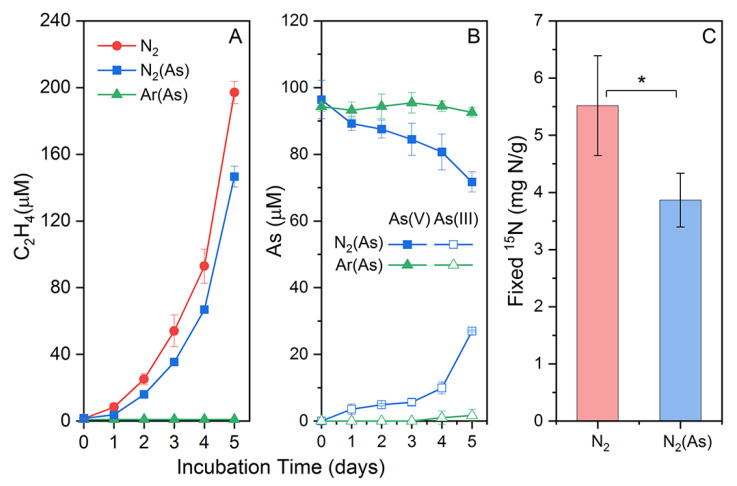
Acetylene accumulation (**A**), As(V) reduction (**B**), and fixed 15N (**C**) during nitrogen fixation of *Paraclostridium* sp. G-11 in Burk’s N-free medium with/without As(V). * indicates a significant difference of fixed ^15^N in Burk’s N-free medium with/without As(V).

*Citrobacter* sp. G-C1 was able to reduce nitrate to nitrite and ammonium during 48 h of anaerobic incubation in CDM medium, using lactate as an electron donor and carbon source (Figure S1). Moreover, the cell growth was coupled with nitrate reduction, indicating *Citrobacter* sp. G-C1 as a dissimilatory nitrate-reducing bacterium. Lactate concentration had distinct effects on the end products of nitrate respiration by *Citrobacter* sp. G-C1. In the presence of 10 mM lactate, 70% of nitrate was reduced to nitrite (55%) and ammonium (15%), while 90% of nitrate was reduced to nitrite (10%) and ammonium (80%) in the case of 20 mM lactate (Figure S1). *Paraclostridium* sp. G-11 was able to fix nitrogen gas, indicated by increasing cumulative ethylene observed during incubation in Burk’s N-free medium (Figure 1A). The ^15^N_2_ assimilation experiment further showed the cumulative fixed ^15^N_2_ was up to 5.51 ± 0.87 mg N/g after a 3-day incubation (Figure 1C).

### 3.2. As(V) Reduction Co-Occurred with DNRA and Nitrogen Fixation

**Figure 2:**
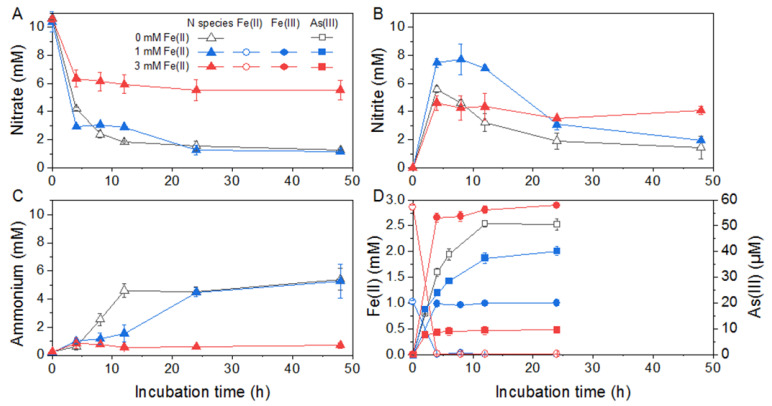
As(V) reduction co-occurred with DNRA by *Citrobacter* sp. G-C1 in CDM medium supplemented with 20 mM lactate, 10 mM nitrate, 100 µM As(V), and different concentrations of Fe(II). (**A**–**C**) refer to concentrations of nitrate, nitrite and ammonium, respectively. (**D**) refers to concentrations of Fe(II) and As(III) during the reaction.

Given the major ammonium production during nitrate respiration, 20 mM lactate was chosen for *Citrobacter* sp. G-C1 as the carbon source and electron donor, to determine the As(V) reduction co-occurring with DNRA. The final concentration of As(V) in the CDM was set to 100 µM, since it had no significant effect on the DNRA of *Citrobacter* sp. G-C1 (Figure S2A) and was about 10-times higher than As concentrations in groundwater in Hetao Plain [16]. The culture experiments showed that 50% of As(V) was simultaneously reduced to As(III) during DNRA (Figure 2). To evaluate the effect of Fe(II) detected in groundwater on As(V) reduction co-occurring with DNRA, different levels of Fe(II) were added to the culture medium. The addition of 1 mM Fe(II) generally had no distinct effect on DNRA, except for slowing the nitrite reduction rate, whereas 3 mM Fe(III) addition strongly influenced the DNRA and As(V) reduction (Figure 2 and Figure S3). It was found that only 40% of nitrate and 8% of As(V) were reduced under the 3 mM Fe(III) condition (Figure 2D).

To test the As(V) reduction co-occurring with nitrogen fixation, we set up a Ar-purged incubation as the control. Differently to the Ar-purged incubation, the N_2_-purged incubation showed an increasing cumulative ethylene and As(V) reduction (Figure 1). The cumulative fixed ^15^N_2_ was up to 3.86 ± 0.47 mg N/g after a 3-day incubation in Burk’s N-free medium (Figure 1C). These results indicated that As(V) was simultaneously reduced to As(III) during nitrogen fixation of *Paraclostridium* sp. G-11. In addition, the As-loaded and N_2_-purged incubation had significantly lower fixed ^15^N and cumulative ethylene contents than the As-free and N_2_-purged incubation (Figure 1C).

### 3.3. Arsenic Demethylation and Methylation

**Figure 3:**
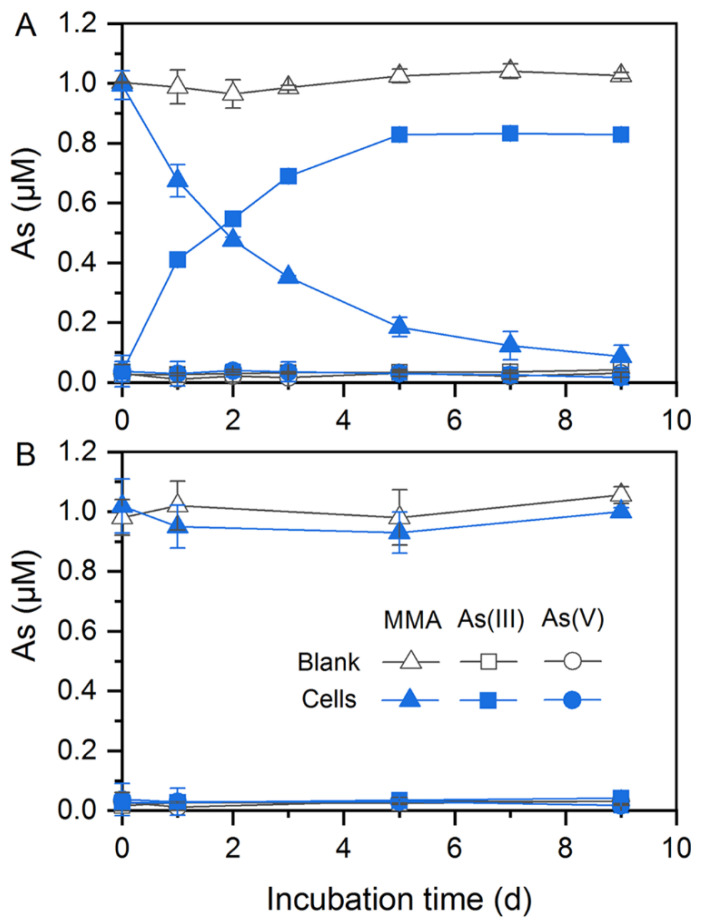
MMA demethylation by *Citrobacter* sp. G-C1 in anaerobic (**A**) and aerobic (**B**) LB medium supplemented with 1 µM MMA.

In addition to As(V) reduction, these two strains could participate in organic As transformation. *Citrobacter* sp. G-C1 was demonstrated to be an As-demethylating bacterium. It could demethylate about 90% and 60% of MMA to inorganic As(III) during a 9-day incubation in anaerobic LB and ST10^−1^ medium, respectively (Figure 3A and Figure S4). The increase in the amount of As(III) coincided with a decrease of MMA in the medium, suggesting that *Citrobacter* sp. G-C1 possesses a two-step pathway of degradation of MMA (first MMA reduction, followed by demethylation) [49,50]. Remarkably, *Citrobacter* sp. G-C1 could not demethylate MMA to inorganic As(III) under aerobic conditions (Figure 3B).

**Figure 4:**
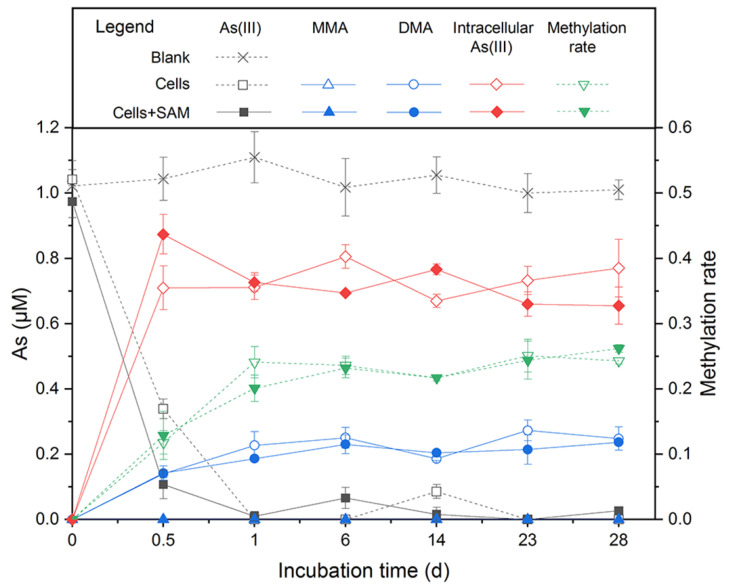
As(III) methylation by *Paraclostridium* sp. G-11 in anaerobic LB medium supplemented with 1 μM As(III). SAM addition was to determine the effect of methyl donor for As biomethylation.

To obtain a closed As balance, we analyzed the As species in the supernatant and the pellet in the As methylation experiment with *Paraclostridium* sp G-11. The results showed that *Paraclostridium* sp. G-11 was a robust As methylator. After an incubation for 6 days, about 0.25 μM, 1.30 μM, and 1.60 μM DMA was found in cell-free supernatants of the medium supplemented by 1 μM, 5 μM, and 10 μM As(III), respectively (Figure 4 and Figure S5). Being the substrate of DMA generation, MMA was detected in cell-free supernatants of 5 μM and 10 μM As(III)-containing medium (about 0.36 μM and 0.60 μM, respectively) (Figure S5). Coupled to a sharp decrease of As(III) in cell-free supernatants during the early stage of incubation, the majority of As(III) entered the cells, thus providing the prerequisite for intracellular As methylation. As(V), MMA, and DMA were not detected in cells, and total As contents were balanced during incubation. The sum of MMA and DMA accounted for 22–36% of the As(III) added in the medium. Furthermore, the addition of a methyl donor (SAM) to the medium did not cause a significant increase of MMA and DMA, as observed in *Shewanella oneidensis* MR-1 [31].

### 3.4. Metabolic Pathways Identified in the Genomes

**Figure 5:**
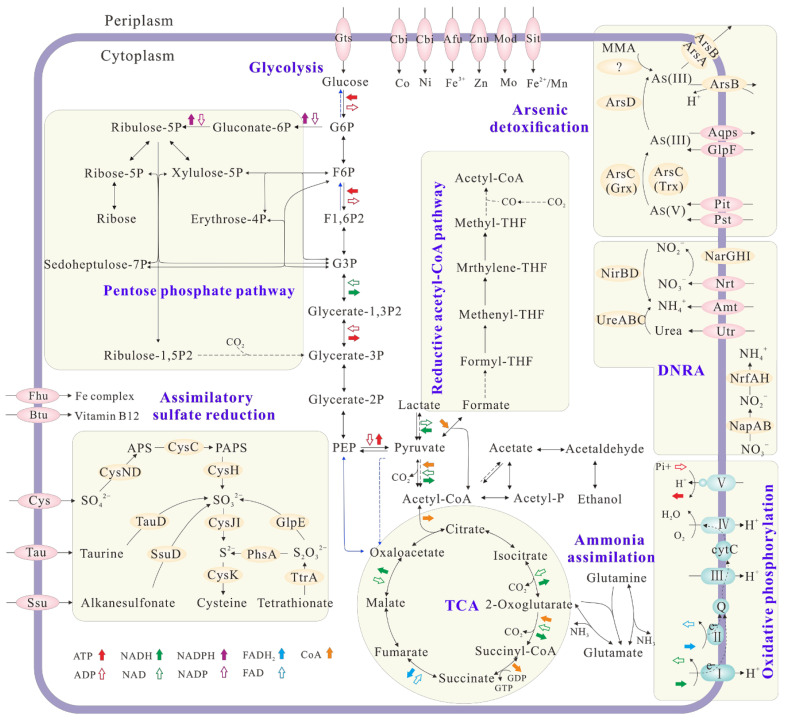
Overview of the metabolic potential of *Citrobacter* sp. G-C1 inferred from the genome functional annotation. Solid and dotted arrows indicate the presence and absence of key functional genes in the KEGG pathways. Genes related to N, As, and S metabolisms and membrane transporters are shown and genes related to C metabolism are omitted.

The draft genome of *Citrobacter* sp. G-C1 consisted of 51 scaffolds, representing an overall 5,187,053 bp, with an average GC content of 51.59% (Table S1). It contained 5214 protein-coding genes, 10 rRNA genes, 78 tRNA genes, and 56 sRNA (small RNA) genes. *Citrobacter* sp. G-C1 possessed a complete set of genes involved in glycolysis, the pentose phosphate pathway, and the tricarboxylic acid (TCA) cycle (Figure 5), which are needed for its heterotrophic activity. The presence of *lldD* (lactate dehydrogenase (cytochrome)) and *acs* (acetyl-CoA synthetase) enabled *Citrobacter* sp. G-C1 to convert lactate to pyruvate and acetyl-CoA, thus supporting cell growth when taking lactate as a carbon source and electron donor (Figure S1). *Citrobacter* sp. G-C1 also indirectly converted acetate to acetyl phosphate and to acetyl-CoA with the combination of *ackA* (acetate kinase) and *pta* (phosphate acetyltransferase). It was noteworthy that *Citrobacter* sp. G-C1 possessed two sets of genes responsible for DNRA: periplasmic *napAB* and *nrfAH*, and cytoplasmic *narGHI* and *nirBD*. As-resistant genes including *arsC*, *arsD*, *arsB,* and *arsA,* rather than the respiratory *arrAB* gene, were identified in the genome, evidencing that *Citrobacter* sp. G-C1 was an As-resistant bacterium. Remarkably, the homologs of known *arsI* genes encoding organic As demethylation were not identified in the genome. In addition, *Citrobacter* sp. G-C1 had a complete set of genes (*cysND*, *cysC*, *cysH*, *cysJH,* and *cysK*) to assimilate sulfate, as well as other organic sulfur compounds, such as taurine, alkanesulfonate, and tetrathionate.

The draft genome of *Paraclostridium* sp. G-11 consisted of 55 scaffolds, representing an overall 3,537,600 bp, with an average GC content of 28.22% (Table S1). Its genome contained 3432 protein-coding genes, 16 rRNA genes, 62 tRNA genes, and 1 sRNA gene. *Paraclostridium* sp. G-11 possessed a set of genes encoding glycolysis and the pentose phosphate pathway, and partial genes responsible for the TCA cycle (Figure S6). The presence of *idh* (lactate dehydrogenase) in the genome showed that *Paraclostridium* sp. G-11 may perform lactic acid fermentation. *Paraclostridium* sp. G-11 could also indirectly convert acetate to acetyl phosphate to acetyl-CoA by *ackA* (acetate kinase) and *pta* (phosphate acetyltransferase). A set of genes involved in the reductive acetyl-CoA pathway were identified in the genome, suggesting its potential for carbon fixation under oligotrophic conditions. *Paraclostridium* sp. G-11 possess a *nif* operon, which probably corresponds to the nitrogen fixation observed in Burk’s N-free medium. Similarly to *Citrobacter* sp. G-C1, As-resistant genes including *arsC*, *arsD*, *arsB,* and *arsA* rather than respiratory *arrAB* gene were found in the genome, indicating that *Paraclostridium* sp. G-11 was also an As-resistant bacterium. Furthermore, the *arsM* gene encoding for As(III) S-adenosylmethionine methyltransferase in the genome of *Paraclostridium* sp. G-11 is responsible for the As(III) methylation (Figure 4 and Figure S5).

## 4. Discussions

Nitrate reduction to ammonium coupling with the cell growth indicated that *Citrobacter* sp. G-C1 is a dissimilatory nitrate-reducing bacterium (Figure S1). In its genome, we identified two sets of genes responsible for DNRA: periplasmic *napAB* and *nrfAH*, and cytoplasmic *narGHI* and *nirBD* (Figure 5). NapAB and NarGHI may mediate the reduction from nitrate to nitrite, and NrfAH and NirBD further reduce nitrite to ammonium, as shown in Figure S1. A high concentration of lactate facilitated nitrate reduction to ammonium, which was attributable to the presence of more electron donors (Figure S1). However, given the stoichiometry of lactate oxidation coupled with nitrate reduction, the lactate added in this study should not be fully oxidized to CO_2_, but may form other intermediates, such as propionate, ethanol, and formate [51]. When cultured with 100 µM As(V), this strain transformed 50% of As(V) to As(III) during DNRA (Figure 2). The co-occurrence of As(V) reduction and DNRA suggested that the As(V) reduction of *Citrobacter* sp. G-C1 was used for detoxification rather than dissimilatory respiration, since nitrate has been demonstrated to repress the As(V) respiratory reductase [12,52]. This was further supported by *ars* operons rather than respiratory *arrAB* genes being identified in the genome (Figure 5 and Figure S7). Compared to the incubation without nitrate addition, the As(V) reduction rate during DNRA was significantly elevated (Figure S2B). As(V) reduction was accompanied by DNRA in the early stages of incubation, suggesting the reduced equivalents from vigorous nitrate respiration, such as the reduced thioredoxin or glutaredoxin, were channeled off to facilitate the As(V)-detoxifying reduction [53]. The presence of a high level of Fe(II) could significantly decrease the extent of nitrate and As(V) reduction (Figure 2). The concurrent decline in DNRA and As(V) reduction probably resulted from restricted microbial activities, due to iron oxide encrustations on the cells, evidenced by Fe(II) oxidation during the early stages of incubation (Figure 2D). A similar phenomenon was observed in other denitrifying bacteria (*Acidovorax* sp. strain BoFeN1, *Pseudogulbenkiania* sp. strain 2002 and *Pseudomonas stutzeri* LS-2), where cells were encrusted by forming Fe(III) minerals, leading to an incomplete reduction of nitrate [54,55,56]. In addition, the observed decrease in As(V) reduction in the presence of Fe(II) was also caused by the adsorption or co-precipitation with the formed Fe oxides, supported by the decrease of dissolved total As concentrations during incubation (Figure S8) [57].

The increasing cumulative ethylene and ^15^N_2_ assimilation, which was observed during incubation in Burk’s N-free medium (Figure 1), indicated that *Paraclostridium* sp. G-11 is a nitrogen-fixing bacterium. In its genome, we identified a *nif* operon (Figure S6), which may respond to nitrogen fixation. Given the same *nifHDK* cluster as *Paraclostridium* sp. G-11, some *Paraclostridium bifermentans* spp. including strain MHMC-14, strain DSM 14991, strain WYM, strain BSD2780061688_150302_D6, and strain ATCC 638 may also fix nitrogen gas under ammonium-limited conditions (Figure S9). When cultured with 100 µM As(V), *Paraclostridium* sp. G-11 showed an increasingly cumulative ethylene, As(V) reduction, and ^15^N_2_ assimilation in N_2_-purged incubation. By contrast, ethylene and As(V) reductions were not found in the Ar-purged incubation. Combined with As-detoxifying genes, including *arsC*, *arsD*, *arsB,* and *arsA,* rather than respiratory *arrAB* gene identified in the genome (Figure S6), these results suggested that *Paraclostridium* sp. G-11 could simultaneously use the *ars* operon to reduce As(V) during nitrogen fixation. Compared to the As-free incubation, the addition of As(V) had significantly lower fixed ^15^N and cumulative ethylene contents (Figure 1C). The reduced nitrogen fixation capacity, on the one hand, may have been due to electron–donor competition and the shunting caused by the reduction of As(V). On the other hand, elevated As levels negatively affected the *nifH* gene expression in free-living nitrogen-fixing bacteria, as revealed by qPCR [58]. Collectively, As(V) reduction co-occurred with DNRA using *Citrobacter* sp. G-C1 and nitrogen fixation of *Paraclostridium* sp. G-11 support the previous hypothesis that ammonium-producing bacteria in groundwater are able to transform As(V) to more mobile As(III) during their respective nitrogen-metabolizing processes, and thus contribute to the genesis of As(III) and ammonium observed in groundwater [16,26]. Seasonal changes of aquifer chemistry may affect indigenous microbial metabolisms, such as the reported activities of ammonium-generating bacteria with an *ars* operon in this study, and thus lead to the seasonal fluctuation in As concentration observed in groundwater [59,60,61,62].

In addition to inorganic As(V) reduction, these two strains were able to mediate organic As transformation. *Citrobacter* sp. G-C1 is an As-demethylating bacterium, which could transform 60–90% of MMA to inorganic As(III) under anaerobic LB and ST10^−1^ medium (Figure 3A and Figure S4). To date, all reported As-demethylating bacteria, such as *Streptomyces* sp. MD1, *Bacillus* sp. MD1, *Nostoc* sp. PCC 7120, and *Enterobacter* sp. CZ-1, have conducted aerobic As demethylation using a nonheme iron-dependent dioxygenase with C·As lyase activity [30,49,50,63]. This dioxygenase is encoded by the *ArsI* gene and uses oxygen for cleavage of the C·As bond, in which one oxygen atom from dioxygen is added to the As and the other is added to the carbon [49]. In this study, *Citrobacter* sp. G-C1 could not convert MMA to inorganic As(III) under aerobic conditions (Figure 3B), and the homologs of known *arsI* genes were not identified in the genome (Figure 5), implying that it may harbor a so far not sequenced gene coding for anaerobic As demethylation. It should be noted that the anaerobic As demethylation of *Citrobacter* sp. G-C1 was also different from the reported methanogenic enrichments of paddy soils, which used methylarsenic as methyl substrates to perform demethylation through the methanogenesis pathway [38]. Molecular clock analysis of *arsM* gene showed that As methylation emerged in Archean environments before the rise of atmospheric oxygen, with methylarsenite being a primitive antibiotic [64]. The anaerobic methylarsenic demethylation could enable microorganisms to alleviate methylarsenite stress and bridge the conversion between inorganic and organic As, before the great oxygenation event.

*Paraclostridium* sp G-11 is an As-methylating bacterium, which could transform 22–36% of inorganic As(III) to MMA and DMA under an anaerobic LB medium (Figure 4 and Figure S5). The *arsM* gene encoding for As(III) S-adenosylmethionine methyltransferase identified in the genome should respond to As(III) methylation. This methylation rate of *Paraclostridium* sp. G-11 (22–36%) is significantly higher than those of previously reported anaerobic As methylators, such as *Clostridium* sp. BXM (10%) [33] and *Methanosarcina acetivorans* C2A (13%) [34], but much lower than aerobic As methylators, including *Streptomyces* sp. strain GSRB54 (67%) [65], *Pseudomonas alcaligenes* NBRC14159 (80%) [66], and *Arsenicibacter rosenii* SM-1 (99%) [67]. Collectively, *Citrobacter* sp. G-C1 and *Paraclostridium* sp. G-11 isolated from high As groundwater possessed the capability for As methylation and demethylation, highlighting the transformation between inorganic and organic As in aquifers [27].

## 5. Conclusions

Nitrogen-metabolizing bacteria may participate in As mobilization in groundwater, similarly to iron-reducing and sulfate-reducing bacteria. In this study, we identified the activities of DNRA and nitrogen fixation of two detoxifying As(V)-reducing bacteria, *Citrobacter* sp. G-C1 and *Paraclostridium* sp. G-11 from high As groundwater, and demonstrated that these two ammonium-producing bacteria, not only transform As(V) to more mobile As(III) during DNRA and nitrogen-fixation processes, but also perform As methylation and demethylation. These results highlight the role of ammonium-producing bacteria on As mobilization and the transformation in groundwater, which help to explain the co-occurrence of ammonium and As(III) and the detection of methylarsenic in groundwater around the world. In addition, the ability for As(V) reduction and As(III) methylation gives *Paraclostridium* sp. G-11 potential as a candidate to remediate As-contaminated media through methylation.

## Data Availability

The raw sequencing data of draft genomes of *Citrobacter* sp. G-C1 and *Paraclostridium* sp. G-11 were deposited to the Short Read Archive database at NCBI at https://www.ncbi.nlm.nih.gov/bioproject/PRJNA754837/ (accessed on 15 August 2021).

## Supplementary Materials

The following supporting information can be downloaded at: www.mdpi.com/article/10.3390/ijerph19159606/s1, Figure S1: DNRA of *Citrobacter* sp. G-C1 and its growth in CDM medium supplemented with 10 mM nitrate and different concentrations of lactate; Figure S2: Effect of 100 µM As(V) on DNRA process by *Citrobacter* sp. G-C1 in CDM medium supplemented with 20 mM lactate, 10 mM nitrate, and 100 µM As(V) (A), and effect of DNRA process by *Citrobacter* sp. G-C1 on As(V) reduction in CDM medium supplemented with 20 mM lactate, 100 µM As(V), and 10 mM nitrate (B); Figure S3: Fe(II) effect on DNRA process of *Citrobacter* sp. G-C1 in CDM medium supplemented with 20 mM lactate, 10 mM nitrate, and different concentrations of Fe(II); Figure S4: MMA demethylation by *Citrobacter* sp. G-C1 in anaerobic ST10-1 medium supplemented with 1 µM MMA; Figure S5: As(III) methylation by *Paraclostridium* sp. G-11 in anaerobic LB medium supplemented with 5 μM As(III) (A) and 10 μM As(III) (B); Figure S6: Overview metabolic potential of *Paraclostridium* sp. G-11 inferred from genome functional annotation; Figure S7: The *ars* operon in the *Citrobacter* sp. G-C1 and *Paraclostridium* sp. G-11; Figure S8: The concentrations of dissolved As(V) and As(III) during DNRA process by *Citrobacter* sp. G-C1 in CDM medium supplemented with 20 mM lactate, 10 mM nitrate, 100 µM As(V), and different concentrations of Fe(II); Figure S9: The *nif* operon in the *Paraclostridium* sp. G-11 and *Paraclostridium* spp. deposited in the NCBI genome database on April 27, 2022; Table S1: Genome assembly statistics of *Citrobacter* sp. G-C1 and *Paraclostridium* sp. G-11.
