# Supplementary material for: Arsenic Mobilization and Transformation by Ammonium-Generating Bacteria Isolated from High Arsenic Groundwater in Hetao Plain, China
Source: Int J Environ Res Public Health. 2022 Aug 4;19(15):9606. doi: 10.3390/ijerph19159606 (PMC9368665; doi:10.3390/ijerph19159606)
Supplement: Supplementary file 1 [file ijerph-19-09606-s001.zip › ijerph-1843754-supplementary.pdf]

Supplementary Materials

Arsenic mobilization and transformation by ammonium-generating bacteria isolated from high arsenic groundwater in Hetao Plain, China

Zhou Jiang[1,2], Xin Shen[1], Bo Shi[1], Mengjie Cui[1], Yanhong Wang[2], Ping Li[2*]

[1]School of Environmental Studies, China University of Geosciences, Wuhan, 430074, P.R. China

[2]State Key Laboratory of Biogeology and Environmental Geology, China University of Geosciences, Wuhan, 430074, P.R. China

*** Corresponding author:** pli@cug.edu.cn

Supplementary Figures (S1–S9) and Tables (S1) and pages 11

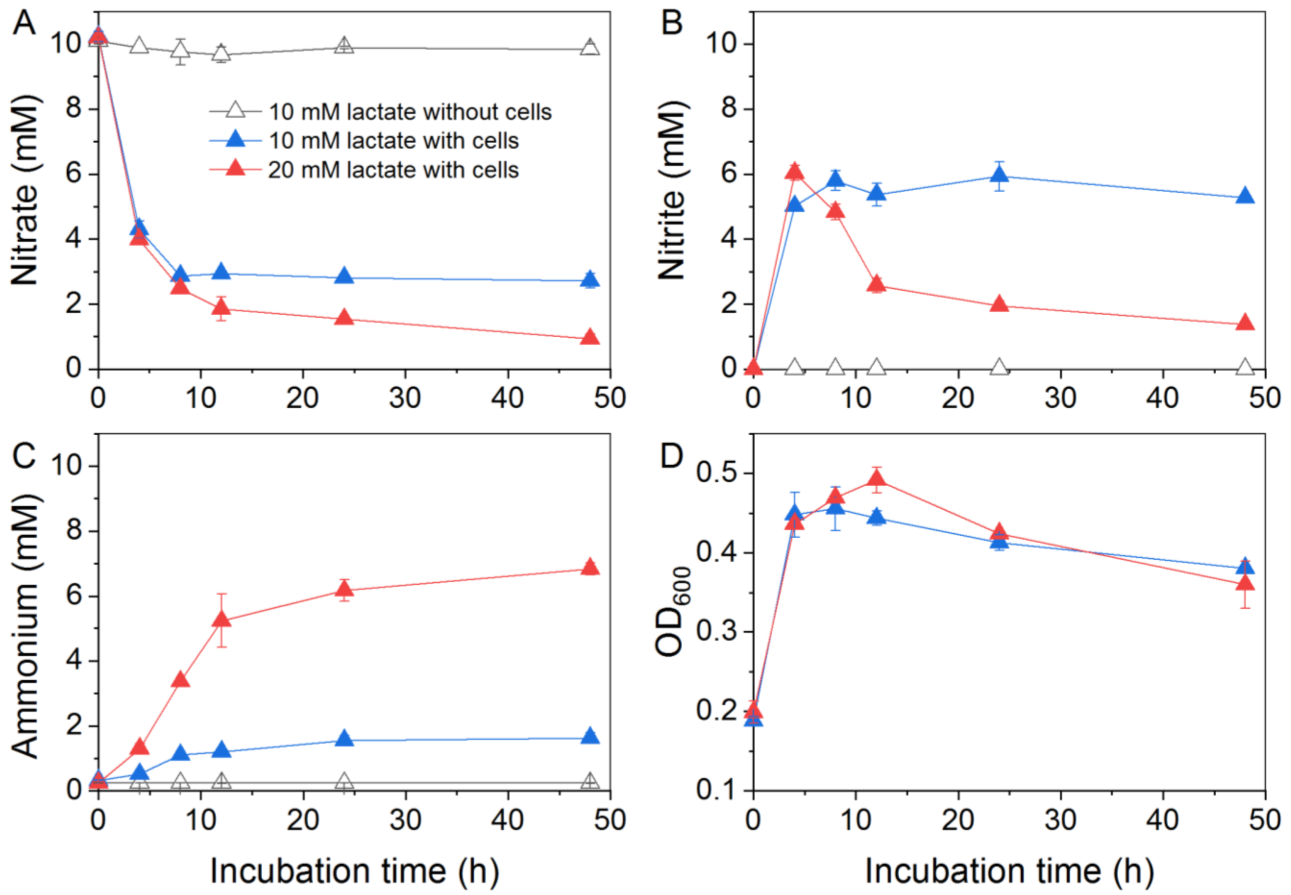

Figure S1. DNRA of *Citrobacter* sp. G-C1 and its growth in CDM medium supplemented with 10 mM nitrate and different concentrations of lactate.

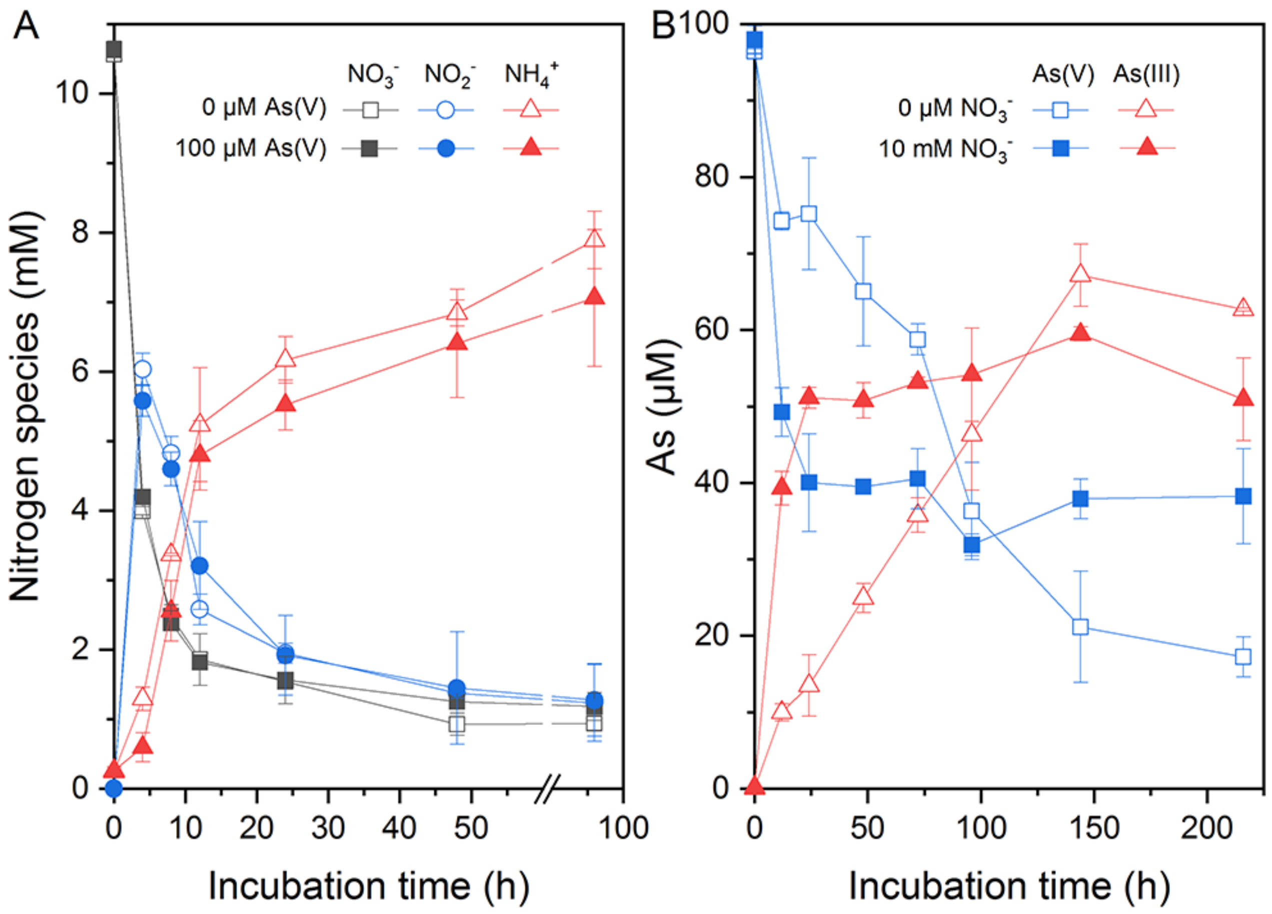

Figure S2. Effect of 100 μM As(V) on DNRA process by *Citrobacter* sp. G-C1 in CDM medium supplemented with 20 mM lactate, 10 mM nitrate and 100 μM As(V) (A) and effect of DNRA process by *Citrobacter* sp. G-C1 on As(V) reduction in CDM medium supplemented with 20 mM lactate, 100 μM As(V) and 10 mM nitrate (B).

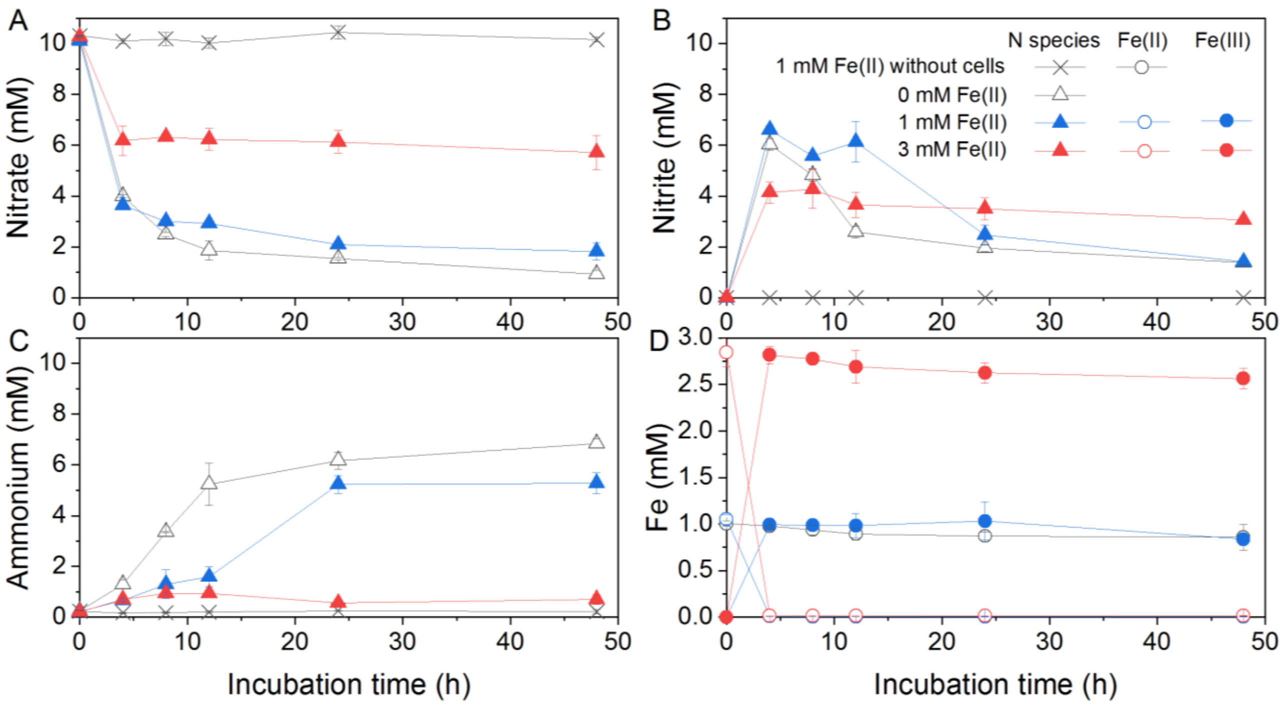

Figure S3. Fe(II) effect on DNRA process by *Citrobacter* sp. G-C1 in CDM medium supplemented with 20 mM lactate, 10 mM nitrate and different concentrations of Fe(II).

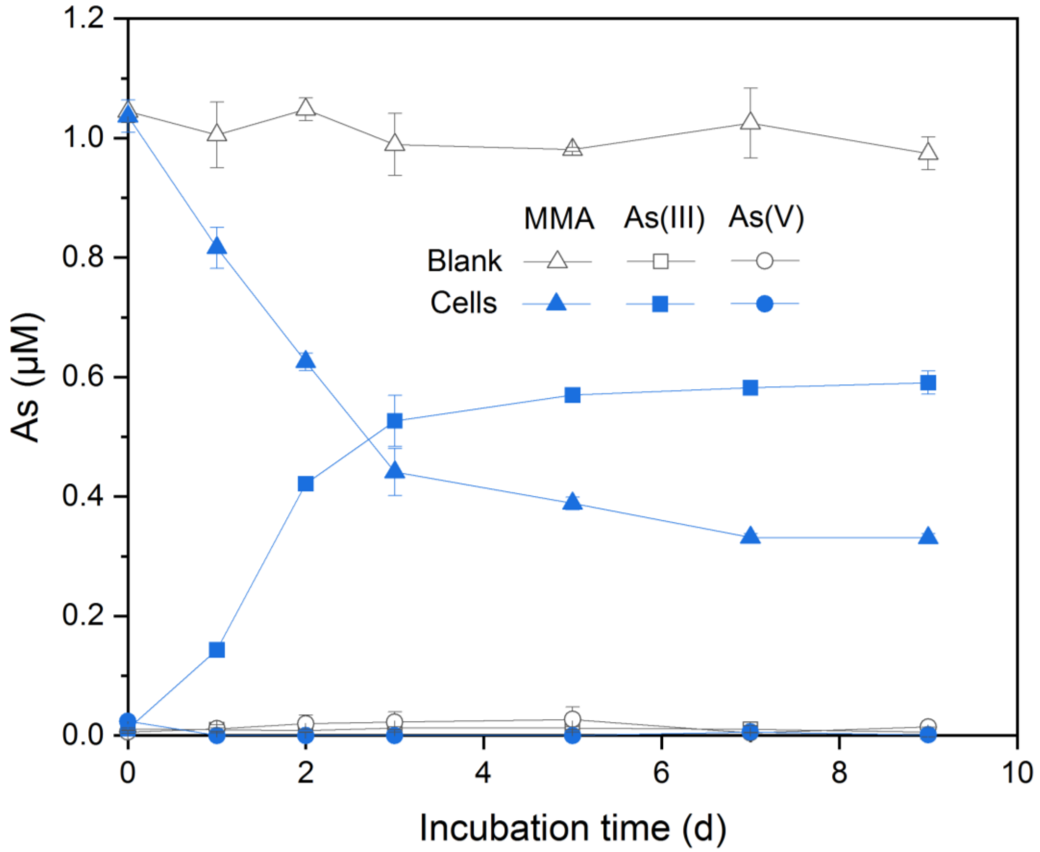

Figure S4. MMA demethylation by *Citrobacter* sp. G-C1 in anaerobic ST10-1 medium supplemented with 1 μM MMA

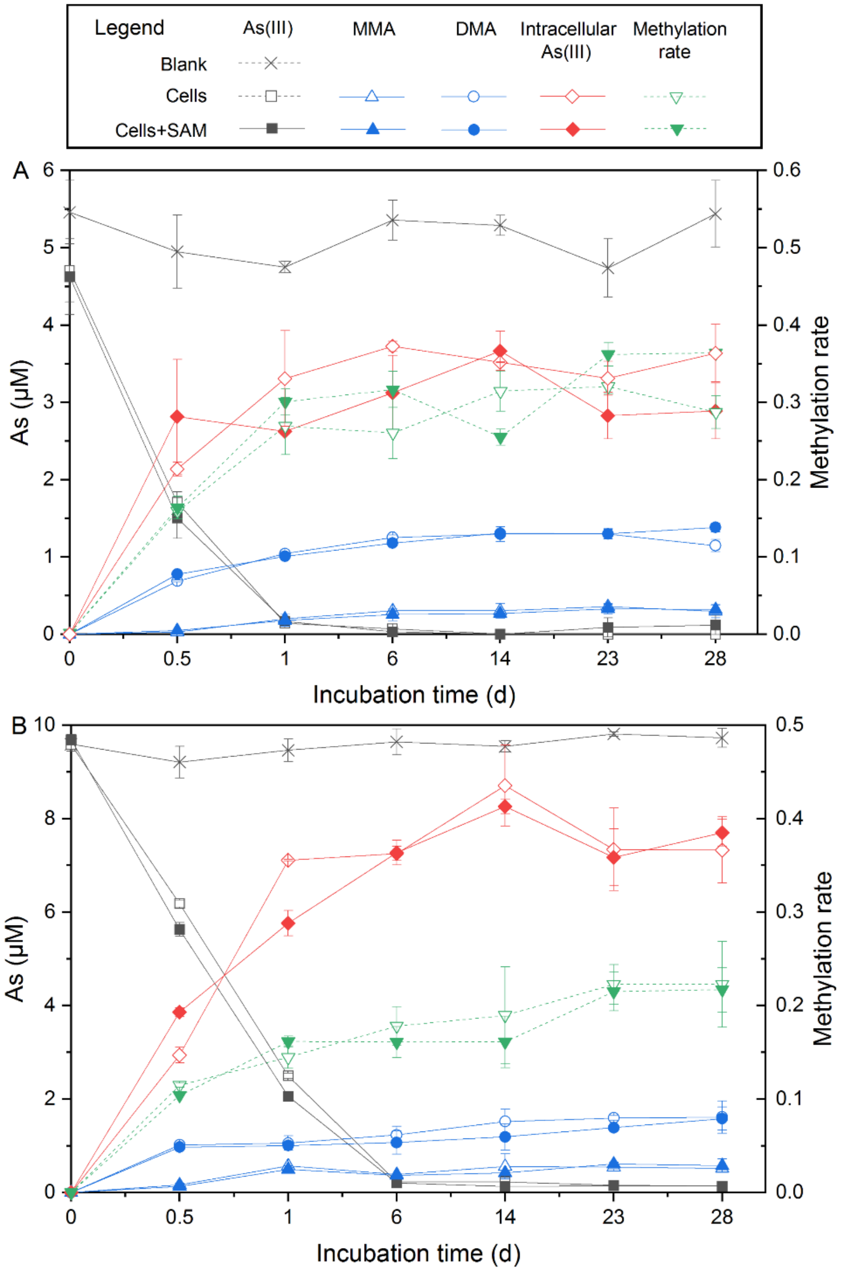

Figure S5. As(III) methylation by *Paraclostridium* sp. G-11 in anaerobic LB medium supplemented with 5 μM As(III) (A) and 10 μM As(III) (B). SAM addition was to determine the effect of methyl donor for As biomethylation.

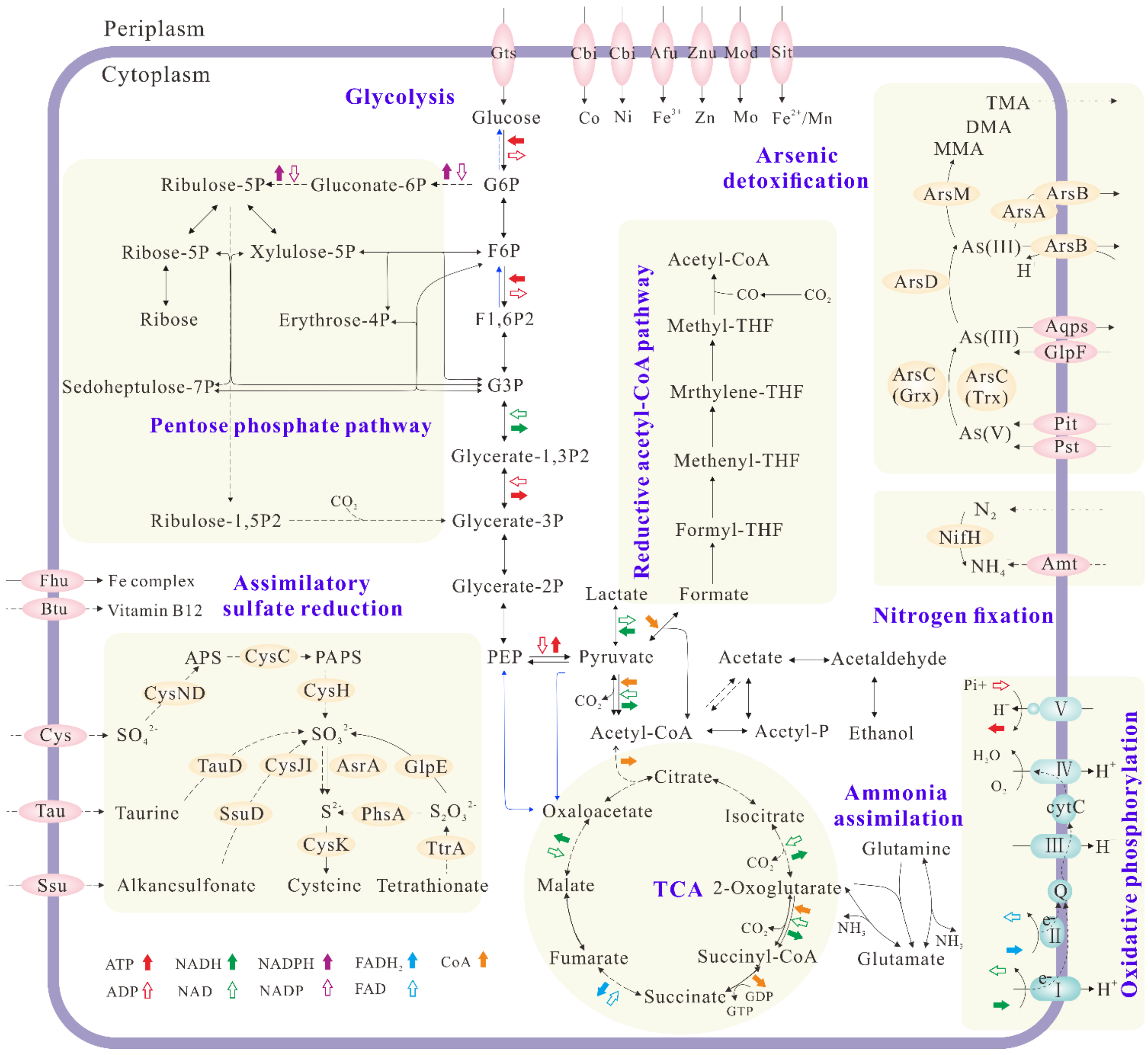

Figure S6. Overview metabolic potential of *Paraclostridium* sp. G-11 inferred from genome functional annotation. Solid and dotted arrows indicate the presence and absence of key functional genes in the KEGG pathways. Genes related to N, As and S metabolisms and membrane transporters were shown and genes related to C metabolism were omitted.

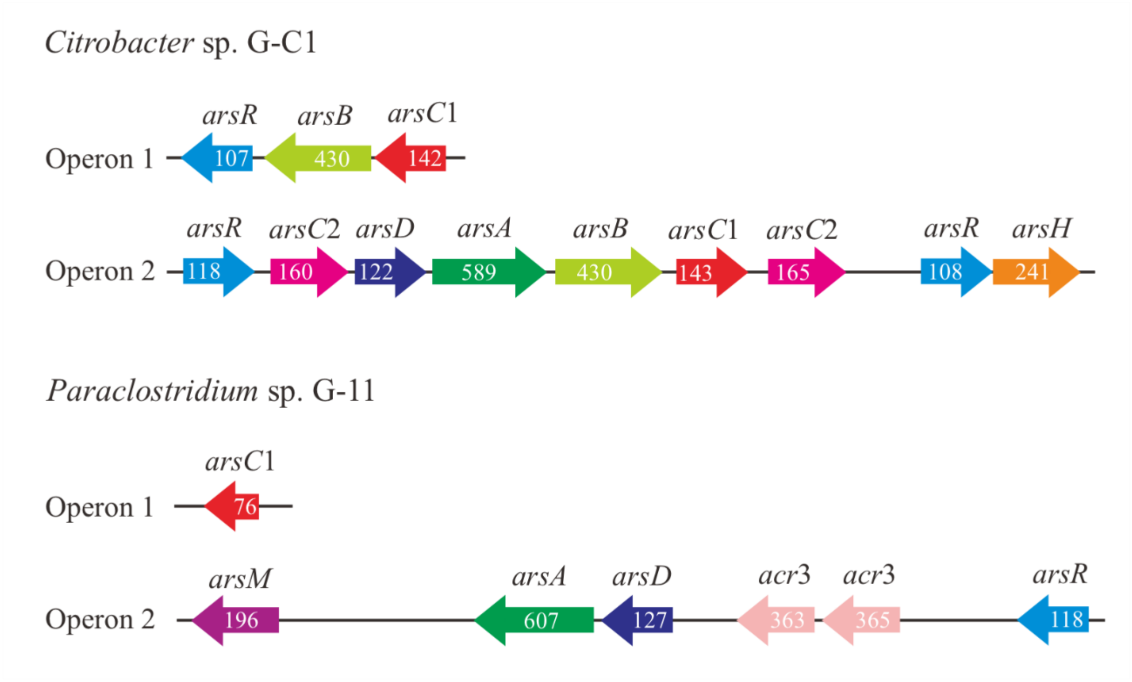

Figure S7. The *ars* operon in the *Citrobacter* sp. G-C1 and *Paraclostridium* sp. G-11. The white number on the arrow shows the length of amino acid sequence. *arsC*1 and *arsC*2 refer to As(V) reductase (glutaredoxin) and As(V) reductase (thioredoxin), respectively.

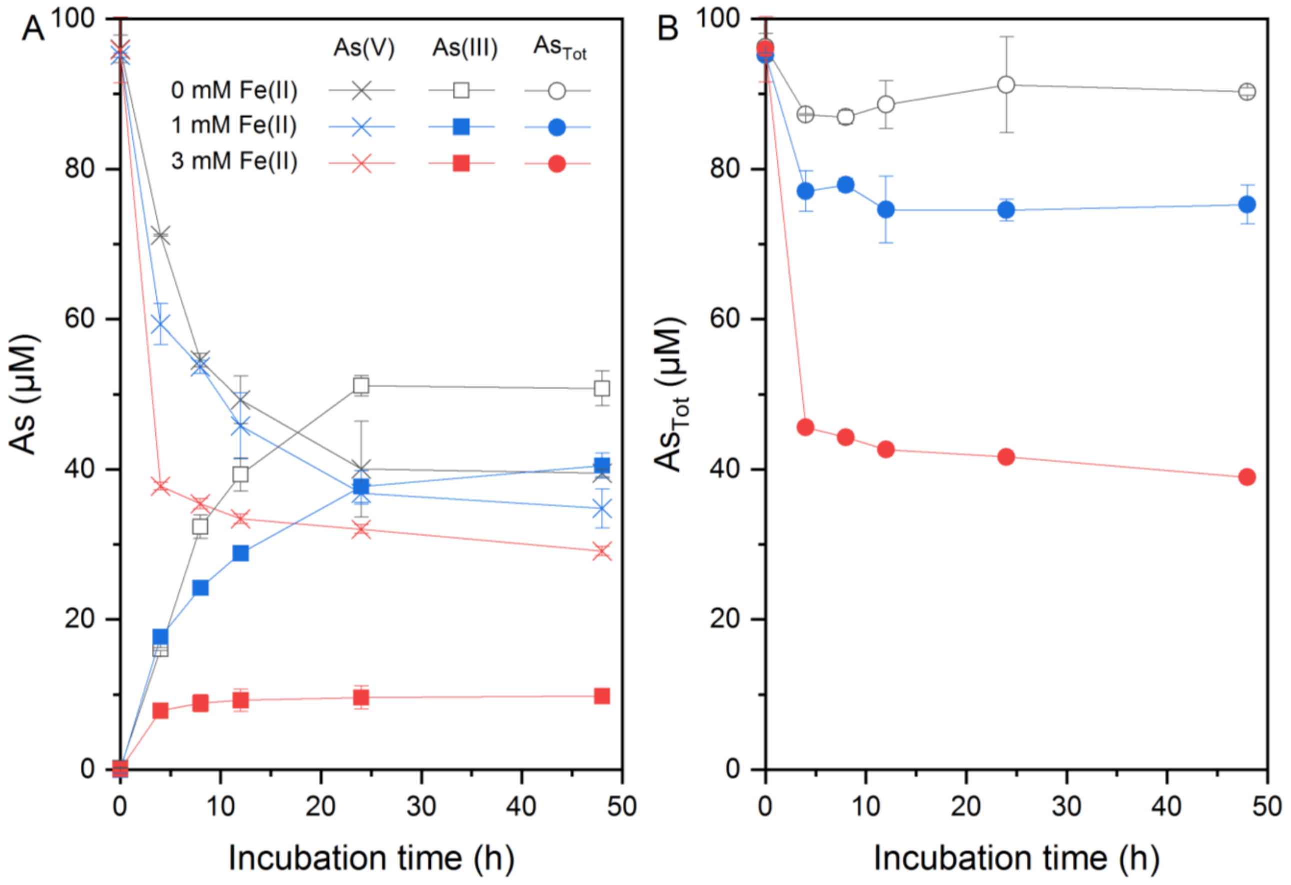

Figure S8. The concentrations of dissolved As(V) and As(III) during DNRA process by *Citrobacter* sp. G-C1 in CDM medium supplemented with 20 mM lactate, 10 mM nitrate, 100 μM As(V) and different concentrations of Fe(II).

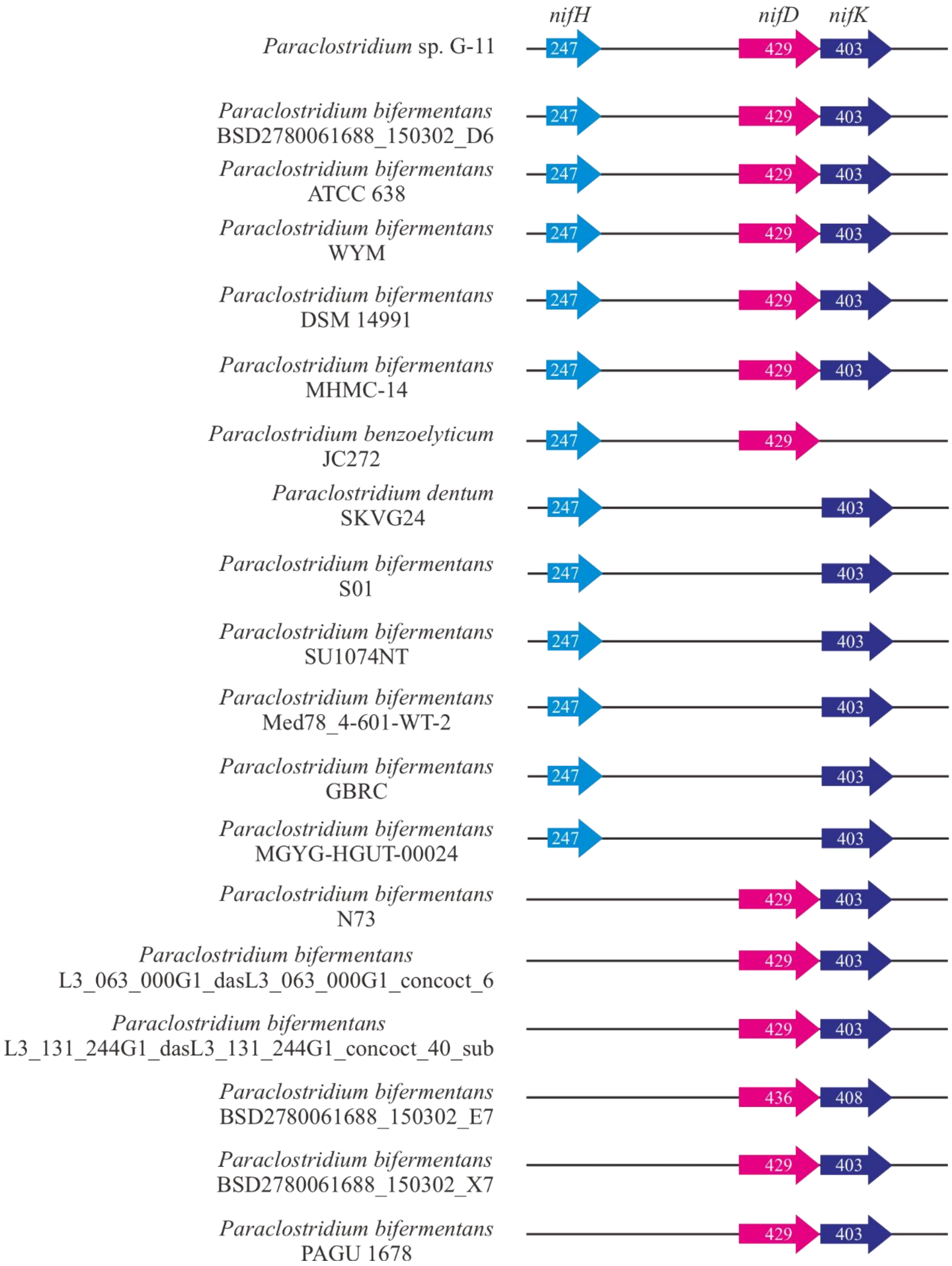

Figure S9. The *nif* operon in the *Paraclostridium* sp. G-11 and *Paraclostridium* spp. deposited in NCBI genome database until April 27, 2022. The white number on the arrow shows the length of amino acid sequence.

Table S1. Genome assembly statistics of *Citrobacter* sp. G-C1 and *Paraclostridium* sp. G-11

Assembly statistics	*Citrobacter* sp. G-C1	*Paraclostridium* sp. G-11
Raw reads number	7,709,834	9,488,958
Raw reads length (bp)	1,156,475,100	1,423,343,700
Clean reads number	7,491,204	9,153,882
Clean reads length (bp)	1,111,278,815	1,350,929,410
Clean reads Q20 (%)	100	100
Clean reads Q30 (%)	96.84	97.54
Clean reads GC (%)	51.00	28
Scaffold total number	51	55
Scaffold total length (bp)	5,187,053	3,537,600
Scaffold average length (bp)	101,707	64,320
Scaffold maximum length (bp)	901,877	920,841
Scaffold shortest length (bp)	509	505
Scaffold N50 length (bp)	499,327	301,435
Scaffold N90 length (bp)	85120	58361
Scaffold GC (%)	51.59	28.22
Genome size (bp)	5187053	3537600
Protein coding genes number from Scaffold	5,214	3,432
Protein coding genes total length (bp)	4,536,630	3,047,082
Protein coding genes average length (bp)	870	888
Protein coding genes length/Genome (%)	87.46	86.13
rRNA number	10	16
tRNA number	78	62
sRNA number	56	1
